# Organized Silica Films Generated by Evaporation-Induced Self-Assembly as Hosts for Iron Oxide Nanoparticles

**DOI:** 10.3390/ma6041467

**Published:** 2013-04-09

**Authors:** Ioanna Andreou, Heinz Amenitsch, Vlassis Likodimos, Polycarpos Falaras, Petros G. Koutsoukos, Epameinondas Leontidis

**Affiliations:** 1 Department of Chemistry, University of Cyprus, P.O. Box 20537, Nicosia 1678, Cyprus; E-Mail: joan10andreou@gmail.com; 2 Institute of Inorganic Chemistry, Graz University of Technology, Stremayergasse 9/4, 8010 Graz, Austria; E-Mail: amenitsch@tugraz.at; 3 Institute of Advanced Materials, Physicochemical Processes, Nanotechnology and Microsystems (IAMPPNM), Division of Physical Chemistry, National Center for Scientific Research “Demokritos”, Aghia Paraskevi Attikis, Athens 153 10, Greece; E-Mails: likodimo@chem.demokritos.gr (V.L.); papi@chem.demokritos.gr (P.F.); 4 Department of Chemical Engineering, University of Patras, P.O. Box 1414, Patras 265 00, Greece; E-Mail: pgk@chemeng.upatras.gr

**Keywords:** oriented mesoporous silica films, grazing incidence X-ray scattering (GISAXS), iron oxide nanoparticles, impregnation, co-precipitation

## Abstract

In this work, we prepared oriented mesoporous thin films of silica on various solid substrates using the pluronic block copolymer P123 as a template. We attempted to insert guest iron oxide (Fe*_x_*O*_y_*) nanoparticles into these films by two different methods: (a) by co-precipitation—where iron precursors are introduced in the synthesis sol before deposition of the silica film—and subsequent oxide production during the film calcination step; (b) by preparing and calcining the silica films first then impregnating them with the iron precursor, obtaining the iron oxide nanoparticles by a second calcination step. We have examined the structural effects of the guest nanoparticles on the silica film structures using grazing incidence X-ray scattering (GISAXS), high-resolution transmission electron spectroscopy (HRTEM), spectroscopic ellipsometry, X-ray photoelectron spectroscopy (XPS), and Raman microscopy. Formation of nanoparticles by co-precipitation may induce substantial changes in the film structure leading, in our adopted process, to the appearance of lamellar ordering in the calcination stage. On the contrary, impregnation-based approaches perturb the film structures much more weakly, but are also less efficient in filling the pores with nanoparticles.

## 1. Introduction

Ordered inorganic mesoporous films are a promising category of functional materials for many applications (e.g., in catalysis, drug delivery, sensing, and photonics) due to their high specific surface area, pore sizes that can be tailored in the nanometer range, and the possibility to incorporate functional groups, or to immobilize active molecules in the mesopores or in the inorganic network [1,2,3,4,5,6,7,8]. A popular solution-based route to synthesis of mesoporous films is evaporation-induced self-assembly (EISA), combined with dip- or spin-coating, using inorganic precursors such as alkoxides, organo-alkoxides, or chlorides, and organic templating agents such as low molecular weight surfactants or amphiphilic block copolymers [9,10,11,12,13]. Silica films, in particular, have been studied widely, since they serve as models to understand the physicochemical phenomena occurring during self-assembly, since it is possible to tune their pore size, pore orientation (parallel or perpendicular to substrate, wormlike, *etc.*) and structure symmetry (cubic, hexagonal, lamellar, *etc.*) by careful tuning of synthesis and calcination parameters [10,12,14,15,16,17,18]. The periodic structure of the films depends strongly on the template used, the removal of which (usually by thermal treatment) often creates strong modifications of the nanostructure. Among several templating molecules, P123, a particular block-copolymer of the Pluronics family, is often used in the production of mesoporous films as it can provide films with lamellar, hexagonal and cubic symmetry, and leads to stable structures up to considerable calcination temperatures [14,15,16,17,18]. The applications of oriented mesoporous silica films can be greatly expanded by inserting substances inside their pores that impart characteristic properties that do not exist in the original structures. For enhanced optical, magnetic, catalytic, or electronic properties a good choice is to insert metal or semiconductor nanoparticles.

In this work we have examined two ways of inserting *iron oxide* (Fe*_x_*O*_y_*) *semiconductor nanoparticles* in P123-templated oriented mesoporous silica films. We focus particularly on iron oxide nanoparticles because these nanomaterials are very useful for photocatalytic applications [19,20,21,22,23], being quite robust in contact with aqueous solutions (a requirement in many such applications). Although the synthesis and characterization of ordered mesoporous SiO_2_ films is very well established, the literature on insertion of oxide nanoparticles in EISA-generated SiO_2_ films is, in fact, limited [24,25,26,27,28,29]. Direct insertion of prefabricated nanoparticles into presynthesized and calcined silica films with relatively small pore sizes is difficult to apply to mesoporous films, and was not considered in this work. There exist several methods to insert guest nanoparticles into mesoporous structures generated by sol-gel processes, but these have largely been evaluated in the case of mesoporous *monoliths* or *powders*. Bronstein mentions seven different methods in her review [30], two of which are quite general and can be implemented in the case of films. ne is to use the metal precursors of the guest oxides—or in certain cases even preformed oxide nanoparticles [31]—in the sol used in the EISA process for film formation. Some quantity of the precursors is then encapsulated in the forming films, especially if they can have some interaction with the template, and upon post-processing—usually thermal treatment for oxides—they form oxide nanoparticles dispersed in the pores of the resulting silica film. The second method is to prepare the silica film, remove the template by calcination, impregnate the film with precursors of the guest, and make a further calcination step to create oxide nanoparticles on the surface and in the pores of the film. The impregnation approach may become more effective if the metal precursor is electrodeposited into the pores [25,26], or if the pores already contain groups that can bind the metal precursor [32]. Impregnation may also be assisted if one inserts groups that interact strongly with the metal precursors into the films [30,32,33], or if repeated impregnation cycles are used [34]. It is not obvious whether co-precipitation or impregnation is better in terms of overall loading of guest nanoparticles in the film, retention of the film structure upon insertion of the guest, and the distribution uniformity of the guest particles in the pores. Methods that may work well for powders may be considerably less effective for films, given the severe diffusion limitations in the latter case. Small precursor loadings, which ensure structure retention in mesoporous powders, may be either insufficient or destructive for mesoporous films. To our knowledge, the literature does not provide clear guidelines for the formation of well-structured nanocomposites with uniform distributions of guest oxide particles in the pores.

The present investigation must be considered as only a first step in the direction of filling this gap, since we have not examined, at this stage, the effect of known, important parameters on silica loading and structure. For example, calcination temperature is of the utmost importance for the stability of mesoporous silica films, but in the present work we have worked only at a single, albeit reasonable, temperature. Likewise, humidity is important to control and may lead to different film architectures that may influence guest nanoparticle insertion. Because of the complexity of the systems, we have chosen to work with a narrow range of parameters in the co-precipitation or impregnation processes, based on existing literature information. We have also carried out a comparison of the two processes of particle insertion, using Fe*_x_*O*_y_* nanoparticles *only* as model guests. Our approach was to examine the structure of mesoporous oriented silica films, generated in similar ways, before and after the insertion of nanoparticles, and to determine and compare the degree of perturbation of the structure imposed by the two different methods. Structural examination was undertaken using grazing incidence X-ray scattering (GISAXS), high resolution transmission electron microscopy (HRTEM), spectroscopic ellipsometry, X-ray photoelectron spectroscopy (XPS), and Raman microscopy. Both one dimensional (1D) and two dimensional (2D) (in-plane) structural periodicity can be examined in detail using GISAXS [35,36]. Raman microscopy may give useful information about the presence of nanoparticle species on the surface and in the pores of the films [37,38]. It also discriminates between different crystalline forms (e.g., hematite and maghemite, or magnetite in the case of iron oxides) [39,40,41,42] and sometimes even the size of the particles, through measured broadenings and shifts of the Raman bands [43]. Spectroscopic ellipsometry provides the thickness and the overall porosity of the films, and some indication of uniformity and orientation [44,45,46,47,48]. XPS verifies the presence, and provides the oxidation states of various elements in the surface layers of the films [49].

The structure of the paper is as follows. In Section 2 we describe in detail the synthetic methods for film production and the protocols for guest nanoparticle introduction in the films. In Section 3 we present our results, grouping them by the method used for nanoparticle insertion in the structures (co-precipitation *vs.* impregnation). Then we discuss the application of the various characterization methods on these films. Finally, Section 4 contains our concluding remarks.

## 2. Experimental Section

### 2.1. Materials

Pluronic P123 (EO_20_PO_70_EO_20_, MW 5800) was bought from Sigma-Aldrich; tetraethyl orthosilicate (TEOS, 98%) was obtained from Fluka or Sigma-Aldrich; hydrofluoric acid (48%), ethanol (>99.9%), and hydrochloric acid (37%) were obtained from Merck; iron nitrate nonahydrate (Fe(NO_3_)_3_·9H_2_O, >99%) was from Acros Organics; and ammonium fluoride (NH_4_F, ACS reagent, >98%) was from Riedel de Haën. All chemicals were used as received.

### 2.2. Substrates for Film Deposition

Silicon wafers (p-doped (100)) were obtained from Jun He Electronic Material Company, China. They were cleaned with a buffer oxide etch solution prepared from a 40 wt %. NH_4_F solution and a 48% HF solution at 6:1 v/v. The wafers were put in the cleaning solution for 20 s, washed with copious amounts of deionized water, dried in air, and cut to pieces of roughly 1 cm × 2 cm. Microscope glass slides were cut to pieces of 1 cm × 2 cm pieces and cleaned using soap, distilled water, and acetone, placed into an ultrasonic bath with acetone for 20 min total, washed again with acetone, kept in acetone, and dried in air before use. FTO (TEC15, Pilkington) was obtained from Xin Yan Technology Ltd., China, and cut to pieces of 1 cm × 2 cm. The FTO substrates were placed into an ultrasonic bath with acetone for 30 min, cleaned with acetone and deionized water, then sonicated for 30 more minutes in deionized water and kept in fresh water. All substrates were used directly, or up to 36 h after being cleaned.

### 2.3. Film Preparation Protocol

Mesoporous silica films were synthesized by dip-coating via evaporation-induced self-assembly (EISA) using, for the most part, a previously described procedure [50], which is a modification of the method originally described by Alberius *et al.* [15]. This procedure uses TEOS, Pluronic-P123, hydrochloric acid, water, and ethanol in a molar ratio of 1:0.00967:0.0012:6:8.7. In a typical film preparation, 1.4 g of P123 were dissolved in 4.0 g of ethanol, then 5.2 g of TEOS were dissolved in a mixed solution containing 2.7 g of 0.04 wt % HCl and 6.0 g of ethanol, and the mixture stirred at room temperature (r.t.) for 15 min. The two solutions were then mixed and stirred at r.t. for 3 h. Thin films were deposited on silicon, glass or FTO substrates by dip coating at room temperature (deposition speed 50 mm/min in the majority of cases, although a few samples were prepared with 5 or 100 mm/min for comparison). Although the environmental humidity strongly influences film quality and structure in the EISA process [51,52], it was not controlled in the present experiments. The deposited films were subsequently aged at r.t. for approximately 24 h. After aging, the films were calcined at 400 °C for 4 h.

### 2.4. Particle Incorporation Methods

(a) The co-precipitation of the Fe precursor during film formation was attempted by adding Fe(NO_3_)_3_·9H_2_O in two different Fe/Si molar ratios (1/50 and 1/100) in the sol without otherwise changing its composition. The subsequent drying and calcination steps were the same as before.

(b) The impregnation of preformed mesoporous silica films with Fe*_x_*O*_y_* nanoparticles was attempted by immersing the films in 0.05 M, or 0.1 M or 0.2 M Fe(NO_3_)_3_·9H_2_O solutions in ethanol at room temperature and stirring for 2 h. The films were then dried under a nitrogen stream and calcined at 400 °C for 4 h to form the oxide nanoparticles.

### 2.5. Film Characterization Methods

**GISAXS measurements** were performed at the Austrian high-flux beamline of the 2 GeV electron storage ring at ELETTRA (Trieste, Italy) [53], using the standard grazing incidence setup. The sample was mounted on a rotation stage with 0.001° resolution (Newport Micro-Controle Spectra-Physics S.A., Evry, France). The incident X-ray beam (8 keV, 1.54 Å) was collimated into a horizontal elongated rectangular shape of 400 μm by 1000 μm. The grazing angle was set close to the critical angle of SiO_2_, and various measurements were performed with the grazing angle increasing up to 3°. The GISAXS images were recorded by an imaging intensified CCD detector X-Ray Gemstar XIDIS (Photonic Science, Millham, UK) having 2048 pixels × 2048 pixels with 2 × 2 and 4 × 4 binning, placed at a distance of 717 mm from the sample. The angular scale in the small angle regime was calibrated with Ag-behenate powder (*d* = 54.376 Å). The CCD images were corrected for spatial distortion and detector non-linearity. Analysis of the 2D patterns, especially respecting horizontal (in-plane), and vertical (out-of-plane) 1D-patterns or “cuts”, was carried out using the program Fit2D.

**Micro-Raman spectra** were measured in backscattering configuration on a Renishaw inVia Reflex microscope using an Ar^+^ ion laser (λ = 514.5 nm, 2.14 eV) and a high-power near infrared (NIR) diode laser (λ = 785 nm, 1.58 eV) as excitation sources. The laser beam was focused onto the samples by means of a 100× objective at power levels of ~0.2 mW/μm^2^. A large number of spectra were acquired from different spots for each studied sample, while the frequency shifts were calibrated by an internal Si reference.

**High-resolution transmission electron microscopy** (HRTEM) examination of selected samples was performed on a JEM 2011 instrument (JEOL), with an accelerating voltage of 200 kV and a point resolution of 0.23 nm. EDS microanalysis was carried out using an INCA *x*-sight Si:Li detector with ultrathin windows (Oxford Instruments). Sample preparation was carried out by scraping pieces of the film from the substrate with a scalpel on a TEM carbon-coated Cu grid.

**Spectroscopic ellipsometry measurements** were carried out on a GES5-SOPRA spectroscopic ellipsometer at a 75° angle of incidence. The spectral region between 250 and 800 nm was scanned at intervals of 10 nm. The optical response of the films was translated into cosΨ and tanΔ functions, from which the dielectric properties of the films were calculated using standard procedures [44,45]. The dielectric functions of both non-calcined and calcined mesoporous silica films were fitted by treating the films as uniform mixtures of SiO_2_ and voids, using the Bruggemann effective medium approximation (BEMA) as shown in Equation (1) [46]:

(1) $$\varphi_{1}\frac{\varepsilon_{1}-\varepsilon }{\varepsilon_{1}+2\varepsilon }+\varphi_{2}\frac{\varepsilon_{2}-\varepsilon }{\varepsilon_{2}+2\varepsilon }=0$$

where ε, ε_1_ and ε_2_ are the dielectric constants of the film, the matrix material (SiO_2_), and the void space respectively at each wavelength used, and φ_1_ and φ_2_ are the volume fractions of SiO_2_ and voids respectively. Deposited nanoparticle layers on the film surfaces were treated as additional layers composed of uniform mixtures of nanoparticle material (e.g., Fe*_x_*O*_y_*) and voids (BEMA approximation) where possible. More complex models have been evoked to examine structures with a dispersion of infiltration of nanoparticles within the pores of the SiO_2_ film, such as triple-layer films with a bottom layer of SiO_2_ and voids and an intermediate layer containing SiO_2_, Fe*_x_*O*_y_* and voids.

**X-ray photoelectron spectroscopy (XPS)** measurements were carried out on a LHS-10 system by SPECS. A Mg anode (MgKα line at 1253.6 eV) was used for X-ray production. The measurements were performed at r.t. and an ultra-high vacuum of 10^−9^ mbar. The transmission energy of 97 eV at the semi-spherical detector gives a linewidth of 0.9 eV for the 3d 5/2 Ag line, and 1.7 eV for the 4f 7/2 Au line. The calibration of the kinetic energy scale was made using the ASTM-E 902-88 standard. The observed electrostatic charging of ca. 0.9 eV was corrected by using the C 1s peak.

## 3. Results and Discussion

### 3.1. Reference Films without Nanoparticle Guests

**Figure 1:**
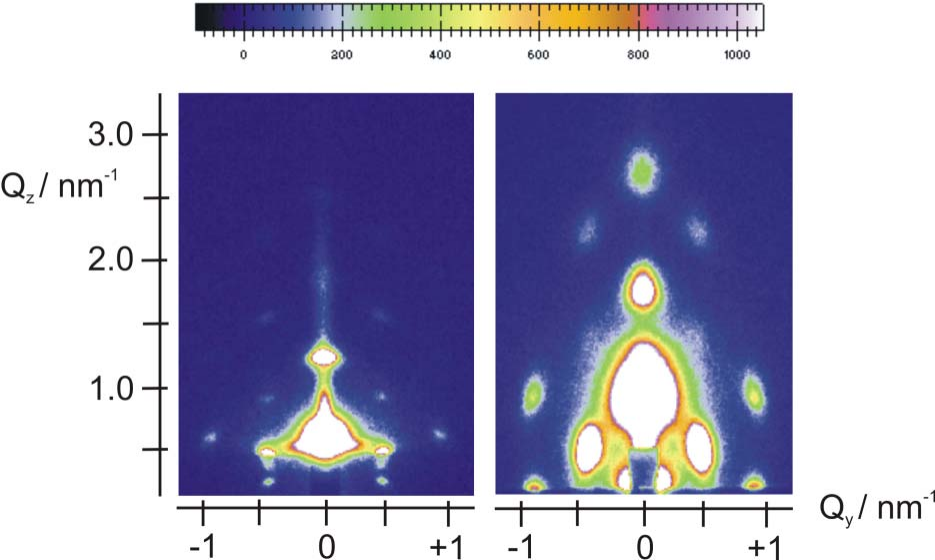
Grazing incidence X-ray scattering (GISAXS) patterns from SiO_2_/P123 films on FTO before (left) and after (right) calcination.

Depending on the synthesis conditions, SiO_2_ films templated by P123 may have a lamellar, hexagonal, or cubic crystalline structure [15,16,17,18]. For a broad range of synthesis conditions, including those of the present work, P123 favors periodic structures with hexagonal symmetry, both before and after calcination [15]. In Figure 1 we compare the 2D GISAXS patterns of a calcined and a non-calcined P123-templated silica film on a FTO substrate. The structure in both cases may be modeled either as a 2D hexagonal (p6mm) or a rectangular lattice (c2mm) [15]. The spacing along the *z*-direction (vertical to the substrate) is equal to 198 ± 4 Å before calcination and 140 ± 6 Å after calcination, in good agreement with the literature [15], showing that a 30% contraction of the original lattice along the *z*-direction has occurred. The in-plane (here the term refers to the *x*-*y* sample plane) dimension on the other hand is not severely distorted upon calcination (the lattice constant is the same within experimental error: 135 ± 6 Å and 130 ± 5 Å). This behavior has often been reported in the literature for SiO_2_ films templated with P123 or other templates [15,54,55,56].

The 2D distorted hexagonal (or rectangular) ordering of the films can be observed in HRTEM images of a calcined film on silicon (Figure S1 in the Electronic Supporting Information section), where periods of 100–130 Å are observed between the cylinders in both directions of the lattice, in rough agreement with the GISAXS pattern.

In Figure S2 we see 1D vertical cuts from samples prepared on glass, FTO and silicon substrates, irradiated at comparable incidence angles. Secondary peaks, mostly for films over glass and FTO substrates, arise due to interfacial reflections [35]. A substrate effect on the film structure definitely exists, although it is not dramatic. D-spacings have been calculated assuming a c2mm lattice, and are tabulated in Table 1. The average d-spacings in the *z*-direction are in the order FTO > Si > glass. This is a systematic result, but the average spacing on FTO is only 6% higher than that on glass. The average in-plane spacings are comparable for the three substrates, those on FTO appearing smaller than the others.

**Table 1:** In-plane and out-of-plane spacings of the c2mm lattices of calcined SiO_2_/P123 films deposited on different substrates. The spacings are averages of 6–7 different films on each substrate.

Substrate	FTO	Silicon	Glass
D (out-of-plane)	139.8 ± 1.3	134.0 ± 1.8	132.0 ± 1.2
D (in-plane)	124.5 ± 1.8	129.1 ± 3.1	128.4 ± 2.1

Spectroscopic ellipsometry was used to obtain thicknesses and porosities of films deposited on Si, using the BEMA approximation. These samples have a thickness in the range of 280 ± 50 nm, in good agreement with literature values [18], and a pore volume fraction of 23% ± 8% (the numbers are averages from several films, the porosity is slightly smaller than those previously reported for P123-templated systems [18], but is known to strongly depend on the thermal treatment).

### 3.2. Insertion of Nanoparticles by Co-Precipitation

**Figure 2:**
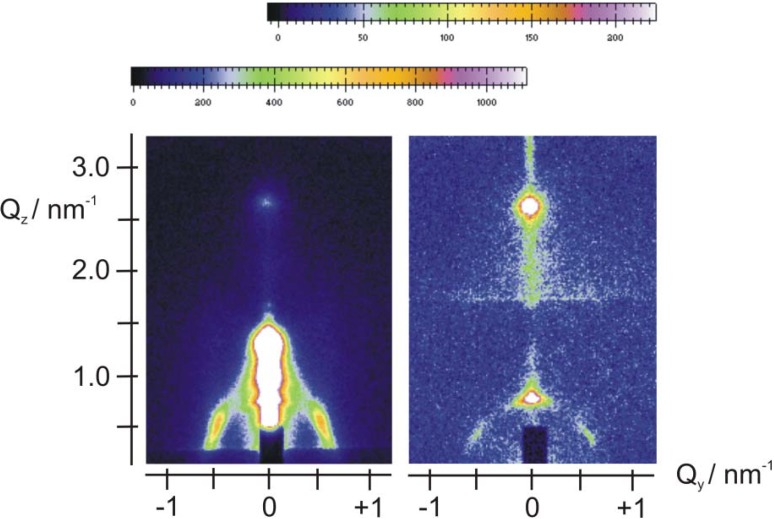
GISAXS patterns of an Fe-containing, calcined film on Si at 0.42° (left) and 2.52° (right) grazing angle. The top color scale refers to the left pattern and the bottom scale to the right pattern.

The insertion of Fe*_x_*O*_y_* nanoparticles was attempted by adding Fe(NO_3_)_3_ in the original sol of the EISA process, depositing the film and generating the oxide particles in the calcination stage. Before calcination the films exhibit the expected, typical hexagonal structure, similar to that observed in Figure 1 (see Figure S3 for a typical pattern of an Fe-containing film before calcination). Typical patterns of a calcined film on Si at two grazing angles (0.42° and 2.52°) are shown in Figure 2.

**Figure 3:**
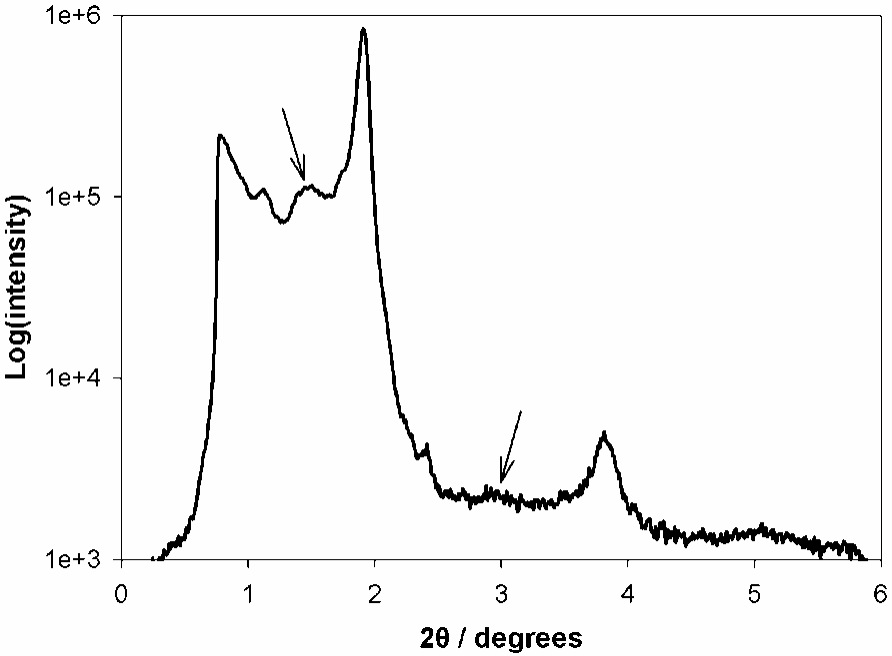
Plot of intensity *vs.* 2*θ* derived from a one dimensional (1D) vertical cut of the GISAXS pattern of an Fe-containing calcined film on Si at a grazing angle of 0.42°.

**Figure 4:**
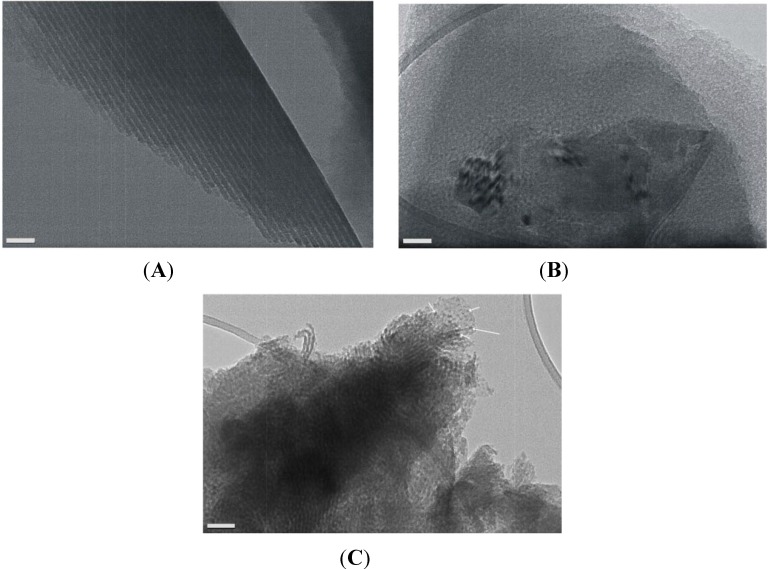
High-resolution transmission electron microscope (HRTEM) images from an Fe-containing calcined sample. (**A**) Lamellar structure with a spacing of 5.0 ± 0.5 Å; (**B**) indication of wormlike domains; (**C**) a domain containing small nanocrystals (indicated by white arrows) inside the pores. The scale bar is 20 nm in (**A**) and 50 nm in (**B**) and (**C**).

Significant differences appear in these patterns with respect to those of Figure 1. There is a ring and the rest of the in-plane structure has largely disappeared. Rings are often associated with the formation of a network of wormlike pores lying mostly parallel to the substrate, or with a broad in-plane distribution of orientations of hexagonal domains [10,54,55,56]. However, the ring in Figure 2 is not displaced to higher *q_z_* values as the incidence angle increases, hence it is a “transmission” artifact—obtained as the X-ray beam grazes the edge of the sample. The structure is further investigated by looking at a 1D-cut (Figure 3) at *q_y_* = 0 for the 0.42° grazing angle, since the 2D pattern does not allow for a clear examination of this line. The main peaks are located at 1.9° and 3.8°, indicating the possible existence of a lamellar structure, since no in-plane peaks are visible in the pattern of Figure 2. This structure has the rather small lamellar spacing of 46 ± 1 Å if the observed peaks are the (001) and (002). A secondary layered structure (peaks at 1.46° and 2.92° indicated by arrows in Figure 3) with a spacing of ca. 60 Å is also barely visible in the 1D-pattern of Figure 3. Characteristic HRTEM images of this sample are provided in Figure 4. Figure 4A suggests the existence of a lamellar phase, with a spacing of 50 ± 5 Å, which is in good agreement with the spacing estimated from the GISAXS pattern. Figure 4B shows that parts of the sample contain wormlike domains. In Figure 4C we see layers with a periodicity of about 60 Å. In addition, Figure 4C reveals the existence of nanoparticles with very small diameters (4–6 nm, comparable to the pore diameters) within the pores of the film. EDS can attest that Fe is present everywhere in this sample, but cannot prove that it is in the form of Fe*_x_*O*_y_* nanoparticles. Because they are so small and their mass fraction in the films is likewise small, their Raman signal was not measurable. Likewise, the XPS spectrum shows very weak Fe^3+^ peaks and no evidence for metallic iron (see Figure S4 in the Electronic Supporting Information Section).

When these samples were examined with spectroscopic ellipsometry, it was found to be possible to model the films as a uniform mixture of SiO_2_ and voids, completely omitting the Fe*_x_*O*_y_* (see Figure S5 in the Electronic Supporting Information Section). The results of the ellipsometric fits are that the thickness of the films on silicon is 270 ± 20 nm and the pore volume fraction is variable, but well below 20%. While film thickness is similar to that of the films that do not contain nanoparticles, the apparent decrease in pore volume must be related to the partial filling of the pores with nanoparticles. We did not detect structural differences between samples obtained with different Fe^III^ concentrations in the original sol, although the final Fe-loading of the films must be different. Overall, these samples pose a considerable challenge: although Fe*_x_*O*_y_* nanoparticles represent only a small part of the total mass and are thus hard to detect, they nonetheless produce a dramatic effect on the film structure after calcination, in the process that we adopted. Li and Lin [27] discuss a very similar method to introduce Fe*_x_*O*_y_* into mesoporous SiO_2_ templated by P123. In their case the crucial drying process was performed in a Petri dish and not on a surface film. However, even in this monolith case, the nanoparticle distribution in the matrix is hard to characterize: the authors could not determine if the particles were in the pores, inside the walls, or at crystallite interfaces [27]. Fornasieri *et al.* [33] introduced CoFe Prussian blue nanoparticles into EISA-generated SiO_2_ films using a mixed co-precipitation-impregnation method. They adopted a different methodology from that used to produce monoliths, in order to produce good quality films with particles in the pores. These examples illustrate how hard it is to introduce nanoparticles in EISA films by adapting methods developed for powders or monoliths.

### 3.3. Insertion of Iron Oxide Nanoparticles into SiO_2_/P123 Films Using Impregnation Methods

Impregnation of porous solids is a popular method to insert nanoparticles or nanoparticle precursors into the pores [28,29,30,32]. The method has the considerable advantage that it uses a well-defined framework for the deposition or creation of nanoparticles, since many inorganic matrices have rigid walls that are not destroyed by the post-treatment. The disadvantages are that the pores may be blocked or impenetrable to the nanoparticles or their precursors, and that the final particle distribution within the matrices may be very inhomogeneous.

**Figure 5:**
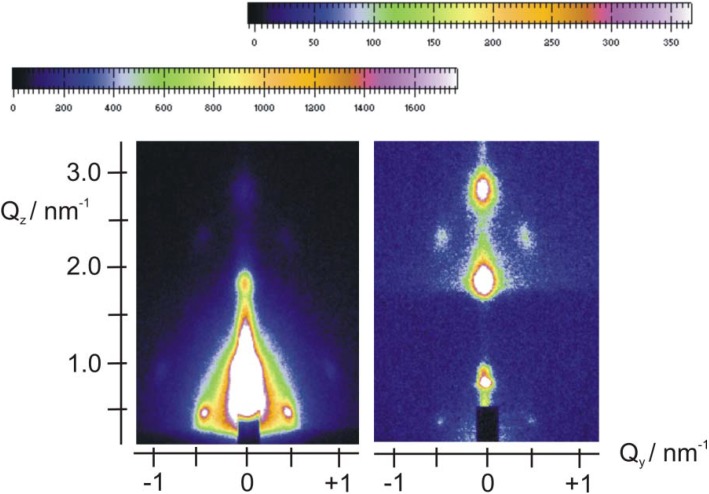
GISAXS patterns of an Fe-impregnated, calcined SiO_2_/P123 film on Si at grazing angles of 0.42° (left) and 2.50° (right). The top color scale refers to the right pattern and the bottom scale to the left pattern.

**Figure 6:**
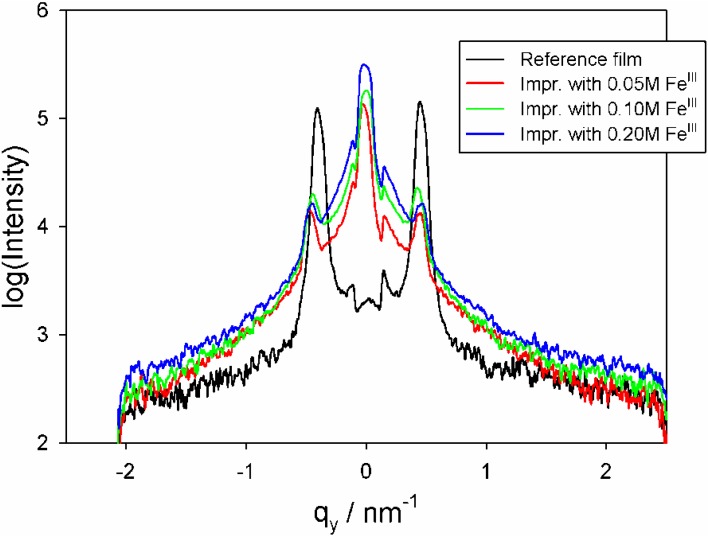
1D horizontal cuts of GISAXS patterns of Fe-containing films on Si, obtained at *q_z_* = 0.51 nm^−1^. A cut for a film not containing Fe is added for comparison.

SiO_2_/P123 hexagonal films were impregnated with Fe^III^ ions using ethanolic Fe(NO_3_)_3_ solutions. 2D GISAXS patterns for a typical sample at two grazing angles (0.45° and 2.50°) are shown in Figure 5. There is an underlying 2D hexagonal lattice, which was observed in the absence of guest nanoparticles (see Figure 1). This lattice is essentially undisturbed, with dimensions b = 133 ± 3 Å, and c = 135 ± 6 Å. Then there is a ring at low *q_z_*, which is an artifact, since it is below the sample horizon in the right hand pattern. Finally, a considerable amount of scattering at small values of *q_y_* is seen, which must be associated, as we shall argue below, with surface deposits of Fe*_x_*O*_y_* nanoparticles. At a grazing angle of 0.45° the in-plane pattern of the 2D hexagonal lattice appears only as a shadow, illustrating the considerable scattering from the surface layer. Figure 6 contains horizontal 1D cuts from films impregnated with different Fe^III^ concentrations and calcined. All of these films were on Si, and the patterns were obtained at similar grazing angles and other GISAXS parameters. The cuts were taken at *q_z_* = 0.51 nm^−1^ and pass through the (11) peak of the c2mm lattice. We see that as the Fe^III^ concentration increases, the scatter at *q_y_* = 0 increases, while the intensity of the (11) peak decreases. This implies that as the Fe^III^ concentration increases a higher mass of particles is deposited on the surface of the films, but also that more iron oxide impregnates the pores, reducing the contrast.

**Figure 7:**
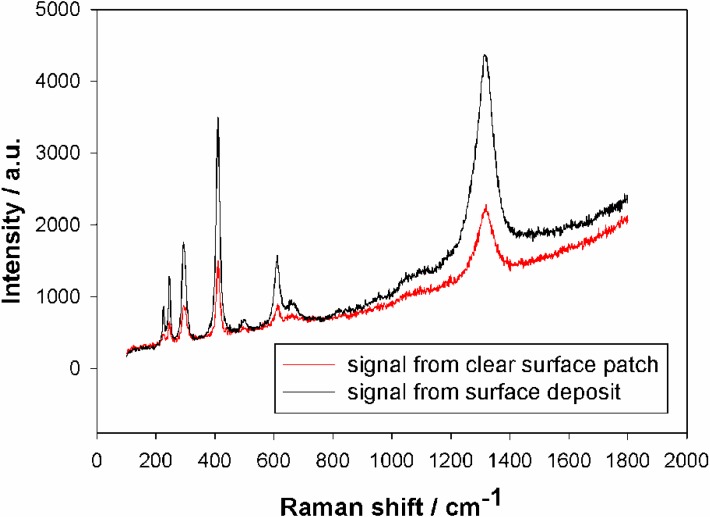
Raman spectra obtained from two positions on an Fe-impregnated sample. The legend indicates the two positions that were sampled. Fe*_x_*O*_y_* surface deposits are clearly visible.

The presence of Fe^III^ in oxide form is proved by the XPS spectrum (see Figure S6 in the Electronic Supporting Information Section), which shows clear Fe^3+^ signals (including even a satellite peak to the Fe 2p 3/2 at 718 eV) and no Fe^2+^ or metallic Fe signals. However, it is not clear if the signal originates from the bulk of the material or from a surface film only. Raman microscopy was used to study the bulk and the surface of this sample, given its complexity. In Figure 7 we see that Fe*_x_*O*_y_* deposits exist on the surface (inset). A series of well-defined Raman bands can be observed, which are identified with the Raman-active modes of *hematite* at 226 (A1g), 246 (Eg), 293 (Eg), 299(Eg), 412 (Eg), 499 (A1g), 612 (Eg) cm^−1^, the disorder-induced mode at ~660 cm^−1^, and its second order overtone at 1318 cm^−1^, verifying the presence of α-Fe_2_O_3_ particles on the SiO_2_ films [38,39]. It is worth noting that the broad mode at ~660 cm^−1^ might be also related to the most intense (A1g) Raman mode of magnetite (Fe_3_O_4_) that occurs at the same frequency [41]. Although the α-Fe_2_O_3_ Raman peaks could be traced on the whole film surface, their intensity varied from spot to spot, as can be seen in the corresponding optical images, indicative of spatial inhomogeneity on the distribution of hematite. The Raman results make a strong case that with this method one deposits Fe*_x_*O*_y_* nanoparticles both on the surface and in the pores of the films. Τhe observation of significant Raman intensity from the α-Fe_2_O_3_ phase, the mass of which is much lower than that of the SiO_2_ substrate, is due to the resonance of the laser excitation energy (2.14 eV) with the hematite band gap (~2.2 eV) (resonance Raman effect) [38]. Similar to a recent publication discussing the insertion of FeCO particles in related films [28], the inserted nanoparticles are very small and hard to locate in the porous structures, but they give a clear Raman signal.

**Figure 8:**
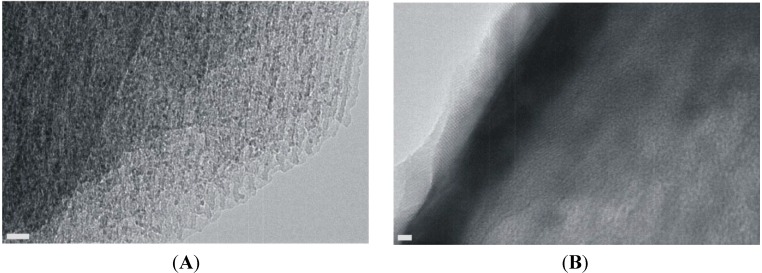
HRTEM images of an Fe-impregnated sample. The scale bar is 20 nm in (**A**) and 2 nm in (**B**).

HRTEM images of samples in this series corroborate (a) that the 2D hexagonal structure is retained—spacings of 100–110 Å can be observed in Figure 8A, in rough agreement with the fit to the GISAXS patterns; (b) that very small iron oxide particles exist in the pores (Figure 8A); and (c) that iron oxide deposits in the form of particles and sheets exist on the surface (Figure 8B).

**Figure 9:**
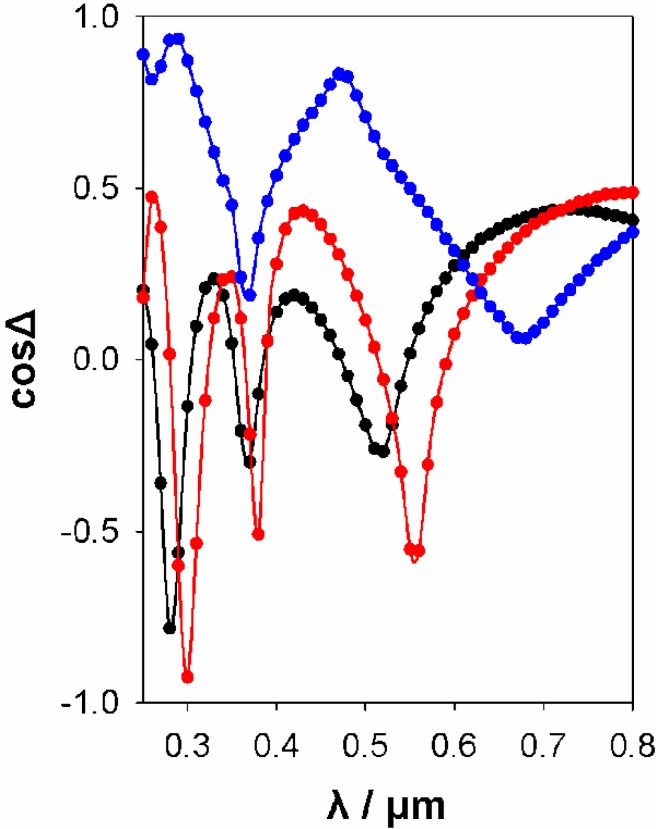
Ellipsometric functions (tanΨ and cosΔ) for a pure SiO_2_ film (black), an Fe-containing film obtained by co-precipitation (red) and an Fe-impregnated film (blue).

Because of the non-uniform surface layers, it is difficult to fit the ellipsometric functions of these samples (see Figure S7 in the Electronic Supporting Information Section for an example of a reasonable fit). Successful fits use a two-layer model: the top layer is a uniform (BEMA) mixture of hematite and voids (typical thickness = 50 nm, typical void fraction = 50%–60%) and the bottom layer is the usual BEMA film of SiO_2_ and voids (typical thickness = 300 nm, typical void fraction = 20%, in excellent agreement with the results in the absence of nanoparticles). A triple layer model, containing an intermediate layer of Fe*_x_*O*_y_*, SiO_2_ and voids, did not improve this fit. A comparison of the ellipsometric functions of three films, one without Fe and the two other with Fe introduced by co-precipitation and impregnation, is given in Figure 9.

The optical functions for the co-precipitated sample and the pure mesoporous silica sample are very similar, while the Fe-impregnated sample appears thicker and different, because of the hematite deposits on its surface. The dimensions and porosities of the three samples are given in Table 2.

**Table 2:** Dimensions and porosities of the films, the ellipsometric functions of which are presented in Figure 10.

Sample type	Thickness/μm	Porosity
Reference SiO_2_/P123 film	0.28	0.23
Fe@ SiO_2_/P123—co-precipitation	0.29	0.18
Fe@ SiO_2_/P123—impregnation	Top layer (hematite + voids) = 0.05	0.58
	Bottom layer (silica + voids) = 0.30	0.19

## 4. Conclusions

The insertion of iron oxide nanoparticles into oriented mesoporous SiO_2_ films (templated with P123) using two different general methods (co-precipitation and impregnation) was examined in this work. Our investigation is not exhaustive, since we used a limited range of synthesis parameters. However these were parameters which, according to the literature, provide good-quality mesoporous silica films of the 2D hexagonal type. The effect of the particles on the film structure obtained after calcination when a co-precipitation method was used was remarkable. The 2D hexagonal structures obtained before calcination are transformed into lamellar structures after calcination, or into mixed structures with lamellar and wormlike domains. Fe*_x_*O*_y_* nanoparticles inserted in the structures are visible with HRTEM, but, because they are very small and have a small mass fraction, do not give measurable Raman signals.

The popular impregnation method gave rather mixed results. The impregnation with Fe^III^ salts was successful and produced films with extensive distribution of small nanoparticles in their pores, but it also resulted in the production of surface deposits, which may hinder the functionality of the films.

In conclusion, the successful insertion of active nanoparticles in silica films to produce a uniform nanocomposite material is not a straightforward task. Many complementary characterization methods and extensive parametric studies are necessary to lay down proper guidelines for the formation of high performance materials for optical and electronic applications.

## Supplementary Materials

Supplementary File 1

## References

[1] Corma A. (1997). From microporous to mesoporous molecular sieve materials and their use in catalysis. Chem. Rev..

[2] Ciesla U., Schüth F. (1999). Ordered mesoporous materials. Microporous Mesoporous Mater..

[3] Hartmann M. (2005). Ordered mesoporous materials for bioadsorption and biocatalysis. Chem. Mater..

[4] Øye G., Glomm W.R., Vrålstad T., Volden S., Magnusson H., Stöcker M., Sjöblom J. (2006). Synthesis, functionalisation and characterisation of mesoporous materials and sol-gel glasses for applications in catalysis, adsorption and photonics. Adv. Colloid Interface Sci..

[5] Wan Y., Zhao D. (2007). On the controllable soft-templating approach to mesoporous silicates. Chem. Rev..

[6] Sanchez C., Boissière C., Grosso D., Laberty C., Nicole L. (2008). Design, synthesis, and properties of inorganic and hybrid thin films having periodically organized nanoporosity. Chem. Mater..

[7] Manzano M., Vallet-Regi M. (2010). New developments in ordered mesoporous materials for drug delivery. J. Mater. Chem..

[8] Ren Y., Ma Z., Bruce P.G. (2012). Ordered mesoporous metal oxides: Synthesis and applications. Chem. Soc. Rev..

[9] Brinker C.J., Lu Y., Sellinger A., Fan H. (1999). Evaporation-induced self-assembly: Nanostructures made easy. Adv. Mater..

[10] Crepaldi E.L., Soler-Illia G.J., Grosso D., Cagnol F., Ribot F., Sanchez C. (2003). Controlled formation of highly organized mesoporous titania thin films: From mesostructured hybrids to mesoporous nanoanatase TiO_2_. J. Am. Chem. Soc..

[11] Gibaud A., Grosso D., Smarsly B., Baptiste A., Bardeau J.F., Babonneau F., Doshi D.A., Chen Z., Brinker C.J., Sanchez C. (2003). Evaporation-controlled self-assembly of silica surfactant mesophases. J. Phys. Chem. B.

[12] Grosso D., Cagnol F., Soler-Illia G.J., Crepaldi E.L., Amenitsch H., Brunet-Bruneau A., Bourgeois A., Sanchez C. (2004). Fundamentals of mesostructuring through evaporation-induced self-assembly. Adv. Funct. Mater..

[13] Grosso D., Boissière C., Nicole L., Sanchez C. (2006). Preparation, treatment and characterisation of nanocrystalline mesoporous ordered layers. J. Sol-Gel Sci. Technol..

[14] Zhao D., Yang P., Melosh N., Feng J., Chmelka B.F., Stucky G.D. (1998). Continuous mesoporous silica films with highly ordered large pore structures. Adv. Mater..

[15] Alberius P.C.A., Frindell K.L., Hayward R.C., Kramer E.J., Stucky G.D., Chmelka B.F. (2002). General predictive syntheses of cubic, hexagonal, and lamellar silica and titania mesostructured thin films. Chem. Mater..

[16] Wei T.-C., Hillhouse H.W. (2007). Mass transport and electrode accessibility through periodic self-assembled nanoporous silica thin films. Langmuir.

[17] Yan M., Dourdain S., Gibaud A. (2008). Analysis of water condensation in P123 templated 2D hexagonal mesoporous silica films by X-ray reflectivity. Thin Solid Films.

[18] Coquil T., Richman E.K., Hutchinson N.J., Tolbert S.H., Pilon L. (2009). Thermal conductivity of cubic and hexagonal mesoporous silica thin films. J. Appl. Phys..

[19] Nozik A.J. (1978). Photoelectrochemistry: Applications to solar energy conversion. Annu. Rev. Phys. Chem..

[20] Scaife D.E. (1980). Oxide semiconductors in photoelectrochemical conversion of solar energy. Solar Energy.

[21] Linsebigler A.L., Lu G., Yates J.T. (1995). Photocatalysis on TiO_2_ surfaces: Principles, mechanisms, and selected results. Chem. Rev..

[22] Hagfeldt A., Grätzel M. (2000). Molecular photovoltaics. Acc. Chem. Res..

[23] Kay A., Cesar I., Grätzel M. (2006). New benchmark for water photooxidation by nanostructured α-Fe_2_O_3_ films. J. Am. Chem. Soc..

[24] Adams W.A., Bakker M.G., Macias T., Jefcoat I.A. (2004). Synthesis and characterization of mesoporous silica films encapsulating titanium dioxide particles: Photodegradation of 2,4-dichlorophenol. J. Hazard. Mater..

[25] Shi K., Peng L.-M., Chen Q., Wang R., Zhou W. (2005). Porous crystalline iron oxide thin films templated by mesoporous silica. Microporous Mesoporous Mater..

[26] Gao F., Naik S.P., Sasaki Y., Okubo T. (2006). Preparation and optical property of nanosized ZnO electrochemically deposited in mesoporous silica films. Thin Solid Films.

[27] Li J., Lin Y. (2008). Facile synthesis of ordered mesoporous silica with high γ-Fe_2_O_3_ loading via sol-gel process. J. Mater. Sci..

[28] Costacurta S., Malfatti L., Innocenzi P., Amenitsch H., Masili A., Corrias A., Casula M.F. (2008). Confined growth of iron cobalt nanocrystals in mesoporous silica thin films: FeCo-SiO_2_ nanocomposites. Microporous Mesoporous Mater..

[29] Krins N., Bass J.D., Julian-Lopez B., Evrar P., Boissiere C., Nicole L., Sanchez C., Amenitsch H., Grosso D. (2011). Mesoporous SiO_2_ thin films containing photoluminescent ZnO nanoparticles and simultaneous SAXS/WAXS/ellipsometry experiments. J. Mater. Chem..

[30] Bronstein L.M. (2003). Nanoparticles made in mesoporous solids. Top. Curr. Chem..

[31] Sundar V.C., Eisler H.J., Bawendi M.G. (2002). Room-temperature, tunable gain media from novel II–VI nanocrystal-titania composite matrices. Adv. Mater..

[32] Zhang W.-H., Shi J.-L., Wang L.-Z., Yan D.-S. (2000). Preparation and characterization of ZnO clusters inside mesoporous silica. Chem. Mater..

[33] Fornasieri G., Aouadi M., Delahaye E., Beaunier P., Durand D., Rivière E., Albouy P.-A., Brisset F., Bleuzen A. (2012). Elaboration of prussian blue analogue/silica nanocomposites: Towards tailor-made nano-scale electronic devices. Materials.

[34] Fröba M., Köhn R., Bouffaud G., Richard O., van Tendeloo G. (1999). Fe_2_O_3_ nanoparticles within mesoporous MCM-48 silica: *In situ* formation and characterization. Chem. Mater..

[35] Tate M.P., Urade V.N., Kowalski J.D., Wei T.-C., Hamilton B.D., Eggiman B.W., Hillhouse H.W. (2006). Simulation and interpretation of 2D diffraction patterns from self-assembled nanostructured films at arbitrary angles of incidence: From grazing incidence (above the critical angle) to transmission perpendicular to the substrate. J. Phys. Chem. B.

[36] Renaud G., Lazzari R., Leroy F. (2009). Probing surface and interface morphology with grazing incidence small angle X-ray scattering. Surf. Sci. Rep..

[37] Huwe H., Fröba M. (2003). Iron (III) oxide nanoparticles within the pore system of mesoporous carbon CMK-1: Intra-pore synthesis and characterization. Microporous Mesoporous Mater..

[38] Kontos A.I., Likodimos V., Stergiopoulos T., Tsoukleris D.S., Falaras P., Rabias I., Papavassiliou G., Kim D., Kunze J., Schmuki P. (2009). Self-organized anodic TiO_2_ nanotube arrays functionalized by iron oxide nanoparticles. Chem. Mater..

[39] De Faria D.L.A., Venâncio Silva S., de Oliveira M.T. (1997). Raman microspectroscopy of some iron oxides and oxyhydroxides. J. Raman Spectrosc..

[40] Gotić M., Ivanda M., Popović S., Musić S., Sekulić A., Turković A., Furić K. (1997). Raman investigation of nanosized TiO_2_. J. Raman Spectrosc..

[41] Shebanova O.N., Lazor P. (2003). Raman spectroscopic study of magnetite (FeFe_2_O_4_): A new assignment for the vibrational spectrum. J. Solid State Chem..

[42] Likodimos V., Stergiopoulos T., Falaras P., Kunze J., Schmuki P. (2008). Phase composition, size, orientation, and antenna effects of self-assembled anodized titania nanotube arrays: A polarized micro-raman investigation. J. Phys. Chem. C.

[43] Choi H.C., Jung Y.M., Kim S.B. (2005). Size effects in the Raman spectra of TiO_2_ nanoparticles. Vib. Spectrosc..

[44] Tompkins H.G., McGahan W.A. (1999). Spectroscopic Ellipsometry and Reflectometry: A User’s Guide.

[45] Fujiwara H. (2007). Spectroscopic Ellipsometry: Principles and Applications.

[46] Aspnes D.E. (1982). Optical properties of thin films. Thin Solid Films.

[47] Braun M.M., Pilon L. (2006). Effective optical properties of non-absorbing nanoporous thin films. Thin Solid Films.

[48] Boissiere C., Grosso D., Lepoutre S., Nicole L., Bruneau A.B., Sanchez C. (2005). Porosity and mechanical properties of mesoporous thin films assessed by environmental ellipsometric porosimetry. Langmuir.

[49] Wertheim G.K., Cheetham A.K., Day P. (1987). X-ray photoelectron spectroscopy and related methods. Solid State Chemistry: Techniques.

[50] Wu C.-W., Yamauchi Y., Ohsuna T., Kuroda K. (2006). Structural study of highly ordered mesoporous silica thin films and replicated Pt nanowires by high-resolution scanning electron microscopy (HRSEM). J. Mater. Chem..

[51] Cagnol F., Grosso D., Soler-Illia G.J., Crepaldi E.L., Babonneau F., Amenitsch H., Sanchez C. (2003). Humidity-controlled mesostructuration in CTAB-templated silica thin film processing. The existence of a modulable steady state. J. Mater. Chem..

[52] Gibaud A., Dourdain S., Gang O., Ocko B.M. (2004). *In situ* grazing incidence small-angle X-ray scattering real-time monitoring of the role of humidity during the structural formation of templated silica thin films. Phys. Rev. B.

[53] Amenitsch H., Rappolt M., Kriechbaum M., Mio H., Laggner P., Bernstorff S. (1998). First performance assessment of the small-angle X-ray scattering beamline at ELETTRA. J. Synchotron Radiat..

[54] Besson S., Gacoin T., Jacquiod C., Ricolleau C., Babonneau D., Boilot J.-P. (2000). Structural study of 3D-hexagonal mesoporous spin-coated sol-gel films. J. Mater. Chem..

[55] Klotz M., Albouy P.-A., Ayral A., Ménager C., Grosso D., van der Lee A., Cabuil V., Babonneau F., Guizard C. (2000). The true structure of hexagonal mesophase-templated silica films as revealed by X-ray scattering: Effects of thermal treatments and of nanoparticle seeding. Chem. Mater..

[56] Besson S., Ricolleau C., Gacoin T., Jacquiod C., Boilot J.-P. (2003). Highly ordered orthorhombic mesoporous silica films. Microporous Mesoporous Mater..

