# Supplementary material for: Organized Silica Films Generated by Evaporation-Induced Self-Assembly as Hosts for Iron Oxide Nanoparticles
Source: Materials (Basel). 2013 Apr 9;6(4):1467–84. doi: 10.3390/ma6041467 (PMC5452312; doi:10.3390/ma6041467)
Supplement: Supplementary File 1 [file materials-06-01467-s001.pdf]

Supporting Information

Figure S1. High-resolution transmission electron microscope (HRTEM) images from a SiO_2/P123 film after calcination. The images show the rectangular (2D-distorted hexagonal) structure of the film. The scale bar is 50 nm in both cases.

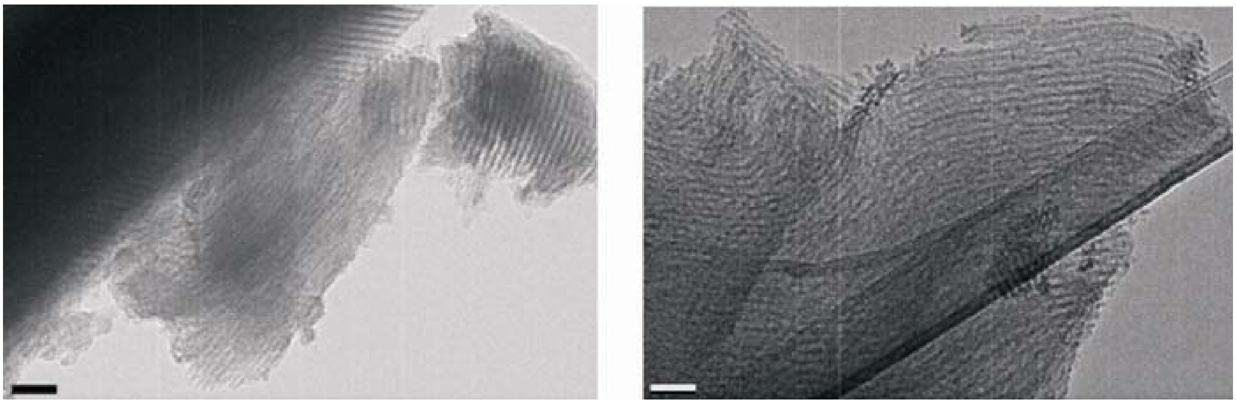

Figure S2. Plots of intensity *vs.* 2*θ* angle, derived from 1D vertical cuts of grazing incidence X-ray scattering (GISAXS) patterns of SiO_2/P123 calcined samples on glass, FTO and silicon.

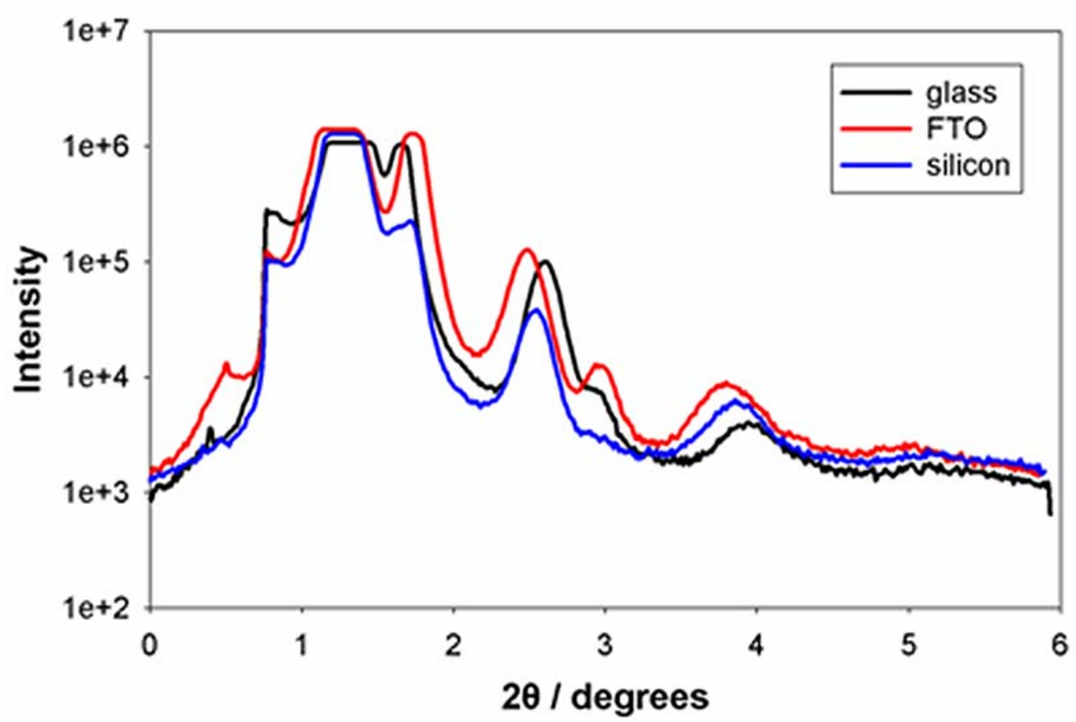

Figure S3. Typical GISAXS pattern of Fe-containing SiO_2/P123 film generated by co-precipitation prior to calcination.

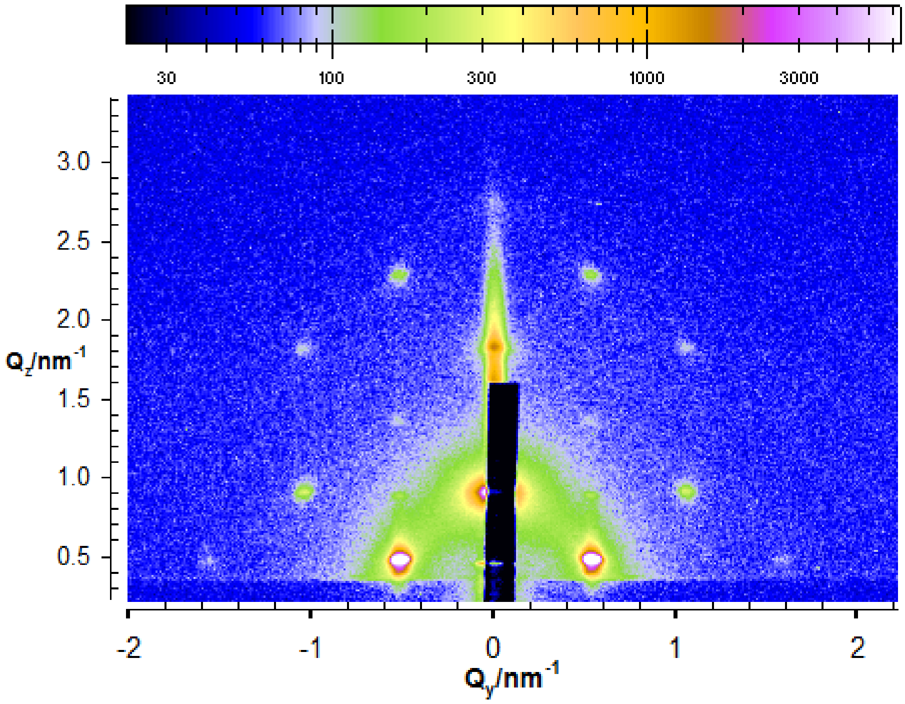

Figure S4. X-ray photoelectron spectroscopy (XPS) spectrum of a Fe-containing SiO_2 film obtained with the method of co-precipitation. The Fe 2p 3/2 and Fe 2p 1/2 bands are barely visible. In the O 1s part of the spectrum there is no evidence for a Fe–O peak.

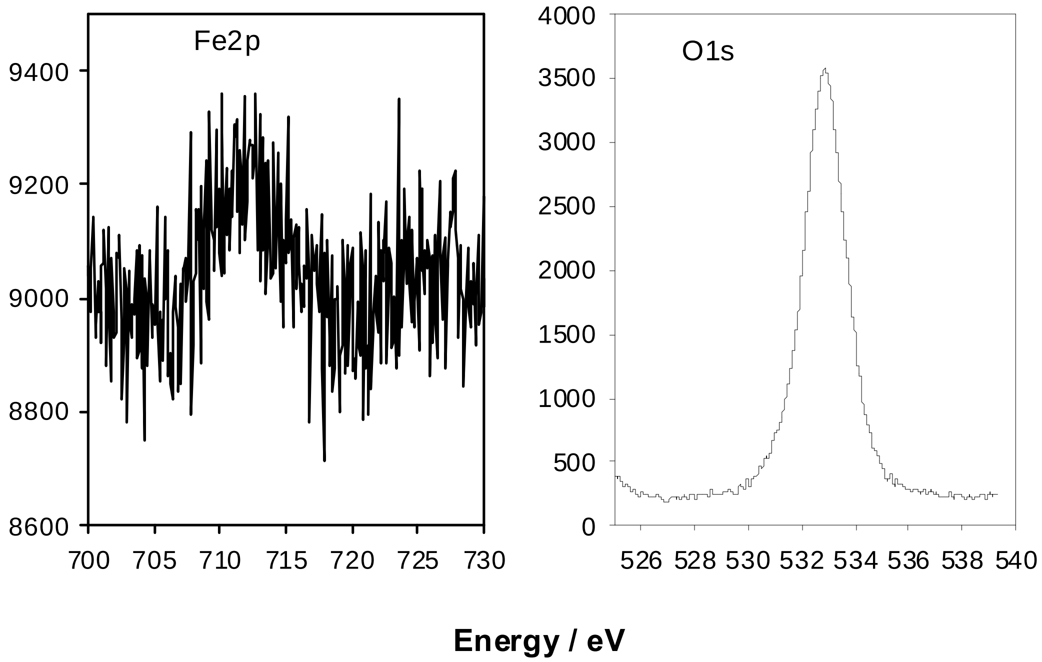

Figure S5. Ellipsometric optical functions of a Fe-containing film obtained from co-precipitation. The functions were fit using a uniform film of SiO_2 and voids in the Bruggemann effective medium approximation (BEMA) approximation and omitting Fe_xO_y. The experimental points (black) are connected with a continuous line, which is a guide to the eye. The red line is the BEMA fit.

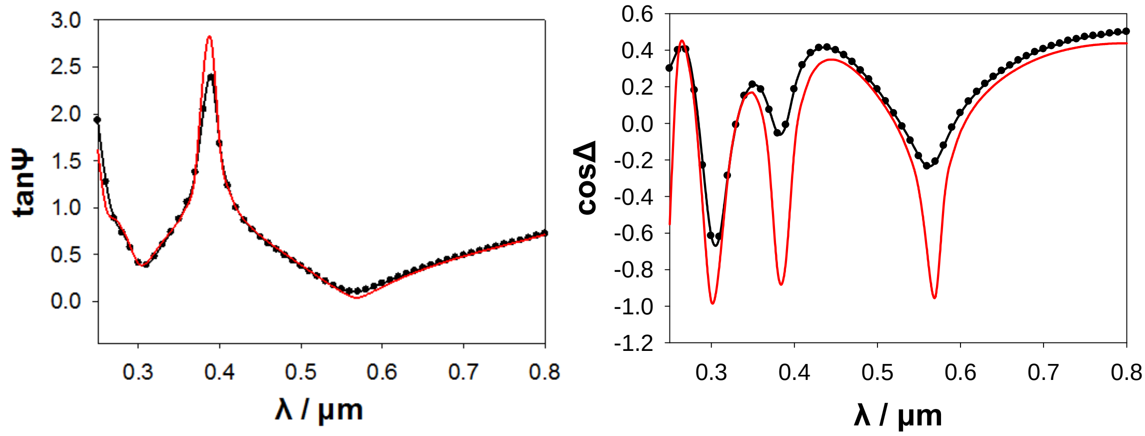

Figure S6. XPS spectrum of a Fe-containing SiO_2 film obtained with the method of impregnation. Left: The Fe 2p 3/2 and Fe 2p 1/2 bands are strong and reveal the presence of Fe^{3+} but not of metallic Fe, or Fe^{2+}. Right: The O1s band can be deconvoluted and reveals the presence of a strong Fe–O band.

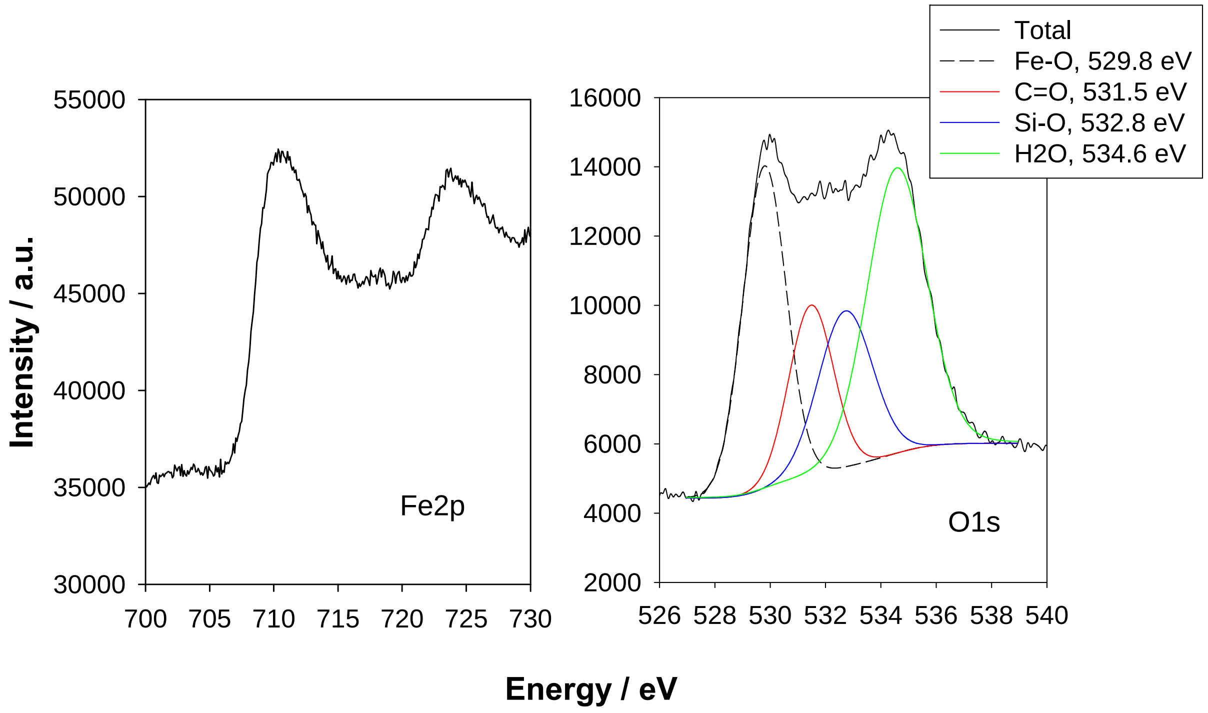

Figure S7. Ellipsometric optical functions of a Fe-containing film obtained by impregnation. The functions were fitted using a uniform film of hematite and voids on the top (BEMA film) and a bottom film of SiO_2 and voids (omitting Fe_xO_y) in the BEMA approximation.

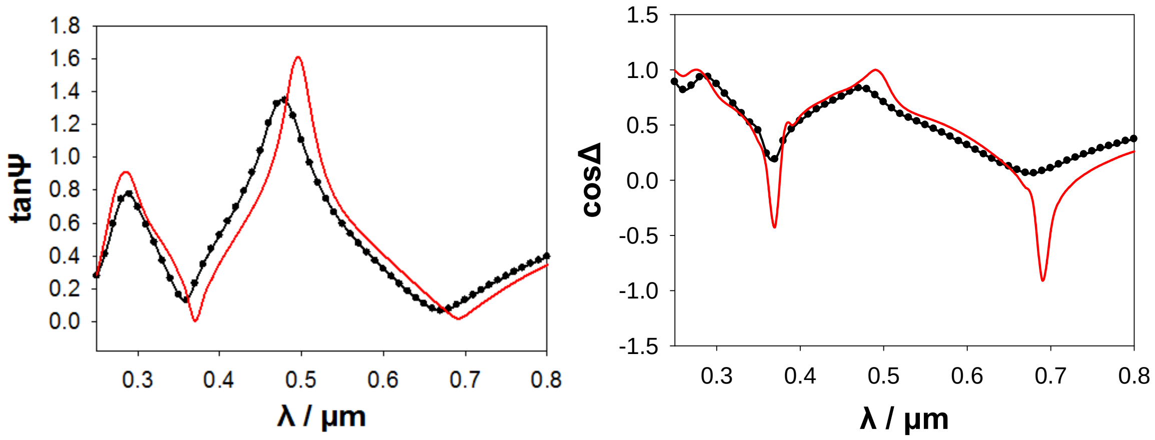

© 2013 by the authors; licensee MDPI, Basel, Switzerland. This article is an open access article distributed under the terms and conditions of the Creative Commons Attribution license (http://creativecommons.org/licenses/by/3.0/).
